# Influence of Different Cooling Rates on Grain Refinement and Tensile Strength of 38MnVS6 Microalloyed Steel after Hot Semiclosed-Die Forging

**DOI:** 10.1021/acsomega.4c10913

**Published:** 2025-02-17

**Authors:** Guilherme O. Goulart, Carlos A. dos Santos

**Affiliations:** School of Technology, Pontifícia Universidade Católica do Rio Grande do Sul - PUCRS, Av. Ipiranga, 6681, Porto Alegre 90619-900, Rio Grande do Sul, Brazil

## Abstract

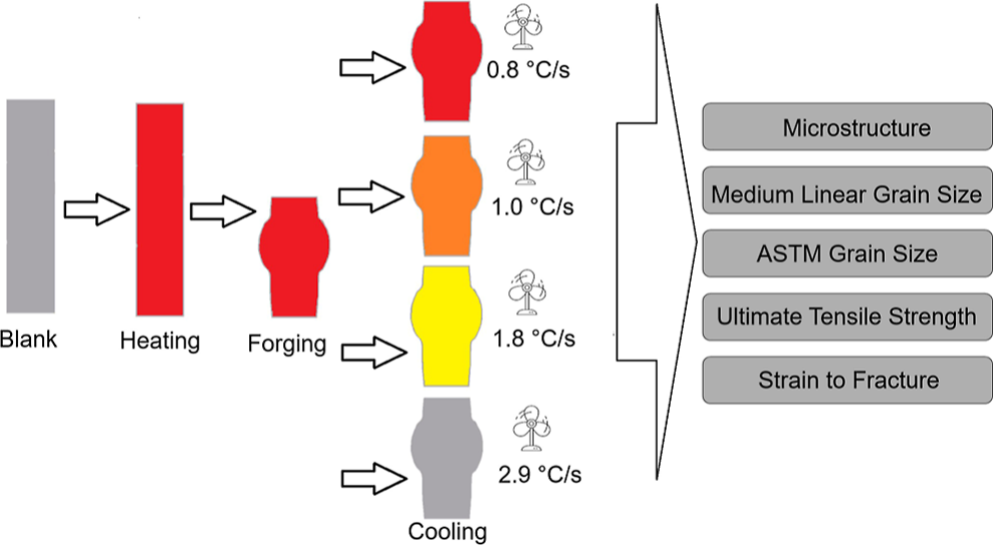

This work investigates the influence of different cooling conditions on the grain refinement and tensile strength of 38MnVS6 microalloyed steel after hot semiclosed-die forging. Samples extracted from cylindrical bars were evaluated in the following conditions: (i) as-received (hot-rolled); (ii) after heating to 1220 °C and cooling at rates of 0.8 °C s^–1^, 1.0 °C s^–1^, 1.8 °C s^–1^, and 2.9 °C s^–1^ using forced air flow (cooled); and (iii) after heating, hot forging, and cooling under the same mentioned conditions (hot-forged). Initially, numerical simulations were carried out to define the specimen dimensions and the die geometry. The hot-forged samples were deformed in an electromechanical press at a strain rate of 0.046 s^–1^. The results showed that the average linear grain length of the as-received material (hot-rolled) was about 68 ± 3 μm. For the cooled samples, the values were 228 and 154 μm for cooling rates of 0.9 °C s^–1^ and 2.9 °C s^–1^, respectively, whereas for the hot-forged condition, the average linear grain length decreased, ranging from 117 μm for samples cooled at 2.9 °C s^–1^ to 131 μm for samples cooled at 0.8 °C s^–1^. Based on the results, higher cooling rates induced higher tensile strength due to grain refinement.

## 1 Introduction

The forging process involves transferring load/energy to a material to be deformed.^1^ One of the advantages of the forging process is the achievement of products with a high level of mechanical properties. For this purpose, several operational parameters need to be controlled to optimize these properties, such as strain rate, preforging geometry, friction behavior, process temperature, and others.^2^ Material characteristics such as chemical composition, microstructure, and mechanical properties should also be considered. It is noted that the ductility in hot forging processes is significantly increased, leading to a reduction in required forming load; however, geometric precision decreases.^3^

Traditionally, the metallurgical industry forges conventional steels for manufacturing; however, such forged products typically require subsequent heat treatments to improve the mechanical strength, increasing processing costs. An alternative to eliminating heat treatments was the addition of alloying elements to steels and the controlled precipitation of carbides and/or carbonitrides with controlled cooling after deformation.^4^ Microalloyed steels or high-strength low-alloy steels (HSLA) are alloys that have a low content of alloying elements in their chemical composition, typically below 0.15%. The objective of this addition is to refine the grain size, increasing the strength, toughness, and hardness. These steels were developed to present yield strength values above 275 MPa, as a function of grain refinement, solid solution formation, precipitation of hardening phases, optimization of texture, and/or phase transformation.^5^

For vanadium microalloyed steels, the formation of carbides explains the increase in mechanical properties. The variation in volumetric fractions, the presence of individual phases, and grain size are controlled by forging temperature parameters, deformation strain, and cooling rate. In general, lower cooling rates result in coarse ferritic-pearlitic structures, ideal cooling rates induce fine ferritic-pearlitic structures, while higher cooling rates form hard microconstituents and phases such as bainite and martensite, respectively.^6−8^ It is noted that for these materials, there are ways to control product characteristics, some of which include process mapping, where forming and cooling parameters are controlled to optimize microstructural formation.^9^ Small additions of vanadium, around 0.13%, are sufficient to alter the CCT diagram, causing Ac_1_ and Ac_3_ to increase by approximately 30 °C.^10^ Experiments have shown that vanadium microalloyed steels associated with controlled air cooling promoted higher hardness, and experiments related to silicon addition, around 1.3%, enhanced the volume of proeutectoid ferrite from 7% to 41%, an enhancement explained by smaller austenitic grain size attributed to undissolved vanadium carbonitrides during austenitization.^11,12^

An important metallurgical feature that needs to be considered is the dynamic recrystallization that occurs during the hot deformation. For example, Łukaszek-Sołek et al.^13^ found that there is a critical deformation value to be exceeded for recrystallization to occur when AISI 4340 steel was analyzed during hot forging. In the same way, thermal changes also significantly affected dynamic recrystallization. From the results, the authors concluded that at 800 °C and a strain rate greater than 0.1 s^–1^, recrystallization did not occur throughout the entire volume of austenite. It was noted that as the strain rate increased, the proportion of precipitated ferrite increased. However, it was observed that it is possible to avoid ferrite precipitation by controlling the hot deformation temperature, since in samples deformed at 900 °C, ferrite was not detected. In general, low cooling rates can cause insufficient hardness due to a high-volume fraction of ferrite and coarse pearlite transformed at high temperatures. On the other hand, high cooling rates can cause the formation of undesired bainite. Tsai et al.^14^ showed for 38MnVS6 steel that the recommended cooling rate window is in the range of 0.5–1.0 °C·s^–1^ when a constant cooling rate is applied. The effect of forging temperature and cooling rate on the metallurgical and mechanical properties of hot-forged 38MnVS6 microalloyed steel was also investigated by Erçayhan and Saklakoğlu.^15^ They found that samples forged at 1050 °C and cooled to 0.75 °C·s^–1^ presented a microstructure composed of coarse pearlite ferrite, with ferrite mainly nucleated at grain boundaries (pro-eutectoid ferrite). When the cooling rate increased to 1.5 °C·s^–1^, the microstructure consisted of pearlite, intergranular ferrite, proeutectoid ferrite, and acicular ferrite. The decrease in the forging temperature and the increase in the cooling rate disturb the formation of the pro-eutectoid ferrite network, resulting in the formation of a refined microstructure formed by pro-eutectoid ferrite (at the grain boundary), polygonal idiomorphic ferrite (intragranular ferrite), and fine pearlite. The aim of this work is to analyze the effect of different cooling rates on the microstructure refinement and tensile strength of 38MnVS6 microalloyed steel after hot semiclosed-die forging. Samples without deformation were also analyzed for comparison purpose.

## 2 Experimental Procedure

The commercial-grade 38MnVS6 (1.1303 steel number) vanadium microalloyed steel was received in the form of 36.51 mm diameter and 6 m long cylindrical bars in the hot-rolled condition. Samples were cut to a length of 120 mm, and the chemical composition was verified by spark optical emission spectrometry (Ametek, SPECTROMAXx) and is listed in Table 1, as well as the European standard EN 10267.^16^ Prior to the hot forging process, numerical analyses were performed to define both specimen and die dimensions according to the imposed loads, deformations, and temperatures.

**Table 1:** Measured and Specified Chemical Composition of the 38MnVS6 Steel (wt %)

element	C	Si	Mn	P	S	V	Cr
measured	0.384	0.56	1.39	0.007	0.023	0.099	0.167
EN 10267	0.34–0.41	0.15–0.80	1.2–1.6	0–0.025	0.02–0.06	0.08–0.2	0–0.3

### 2.1 Numerical Analysis

A pair of forging dies (upper and lower dies) was designed considering the capacity of the electromechanical press and the diameter of the specimens, adopting a compression factor of 2.75 in the controlled region (midlength of the specimen), with an internal cavity of 40 mm in the lower tool (stationary) and 20 mm in the upper tool, resulting in specimens 160 mm long. After deformation, the final length is 100 mm. Based on the premises, heating and forming simulations were conducted using the QForm UK numerical simulation software (Micas Simulations, QForm UK), and the input data are presented in Table 2. Due to the regular geometry of the sample (axisymmetric profile) and the extraction angle of the tools (greater than 3°), lubrication is not necessary.

**Table 2:**
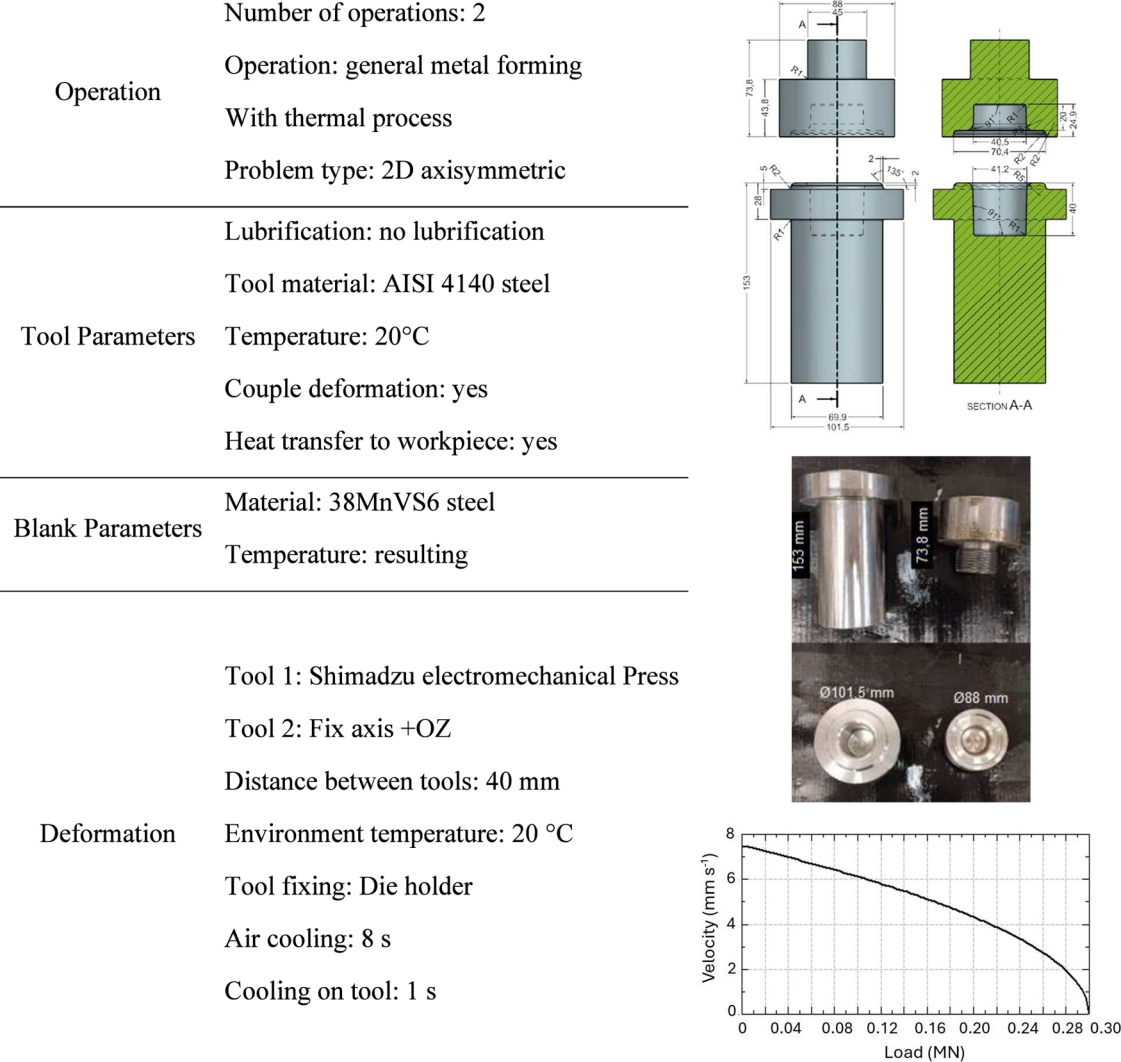
Parameters Used for Designing the Die

The chemical composition of 38MnVS6 steel was set up according to Table 1, and the basic thermophysical properties are from the QForm UK database, as follows: thermal conductivity (*k* = 26.8 W·m^–1^·K^–1^); specific heat (*c* = 620 J·kg^–1^·K^–1^); and density (ρ = 7850 kg·m^–3^ at 0 °C, 7362 kg·m^–3^ at 1300 °C). To predict the behavior of 38MnVS6 steel during hot deformation, the variation in stress (σ) as a function of strain (ε) and temperature (T) was defined according to eq 1
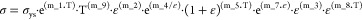
1where the yield strength “σ_ys_” and the constants “m_” are in MPa and are shown in Table 3.

**Table 3:** Constant Values and Variable Ranges of eq 1 [Adapted from QForm UK]

σ_ys_	4241.14		
m_1	–0.00295	m_2	0.38339
m_3	0	m_4	0.00027
m_5	–0.00052	m_7	–0.48576
m_8	0.000143	m_9	0
stress (MPa)	min.: 0.04	max.: 1.25	
strain velocity (mm s^–1^)	min.: 0.001	max.: 500	
temperature (°C)	min.: 800	max.: 1300	

The ranges of variables such as stress, strain velocity, and temperature are also shown in Table 3. These values are valid when established without lubrication, using the Levanov law [QForm UK] with a friction factor of 0.8, a Levanov coefficient of 1.25, a heat transfer coefficient of 50,000 W·m^–2^·K, and a pause coefficient of 0.05. It should be noted that the data presented in Tables 2 and 3 were obtained through the QForm UK software database.

For the tools, the parameters considered for the AISI 4140 steel material were Poisson’s ratio (υ) of 0.29 and yield strength (σ_ys_) of 1020 MPa. The parameters affected by temperature, such as Young’s modulus, thermal conductivity, specific heat, and density are presented in Table 4, with these data obtained from the QForm UK software database, version 10.3.

**Table 4:** AISI 4140 Steel Parameters Affected by Temperature [Adapted from QForm UK]

temperature (°C)	Young’s modulus (MPa)	thermal conductivity (W·m^–1^·K^–1^)	specific heat (J·kg^–1^·K^–1^)	density (kg·m^–3^)
20	210,000	36	375	7716
100	205,000			7692
200			551	7660
300		43		
500	165,000		630	
600		45		
700			975	
800			793	7459
900		46		
1200			663	

### 2.2 Experimental Analysis

For the experiments, an electric resistive furnace (Sanchis Co., 8 kW) was used for heating, with a maximum operating temperature of 1450 °C and temperature control via a type-S thermocouple. The specimens were heated to 1220 °C and kept at this temperature for 28 min (soaking time). A total of 32 specimens were analyzed, with 4 specimens subjected directly to cooling and 28 specimens subjected to hot forging followed by cooling. The forming process was carried out in an electromechanical testing press (Shimadzu AG-IC, 300 kN) using a strain rate of 0.046 s^–1^ (with deformation speed of 7.5 mm·s^–1^).

**Figure 1:**
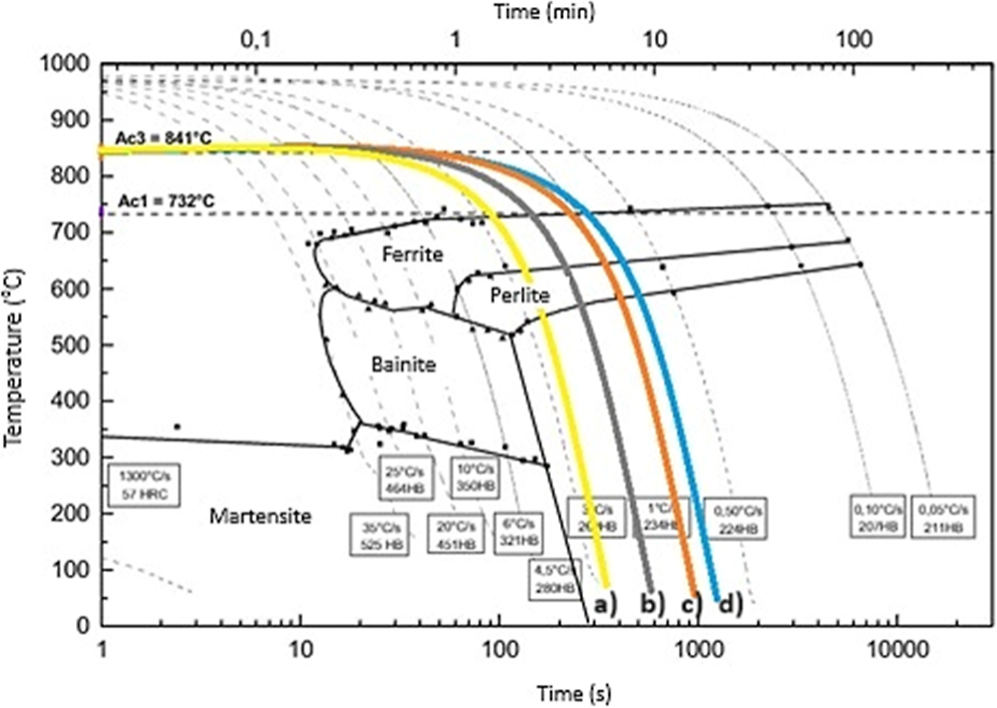
Cooling curves in the CCT diagram of 38MnVS6 steel: (a) 0.8 °C·s^–1^, (b) 1.0 °C·s^–1^, (c) 1.8 °C·s^–1^, and (d) 2.9 °C·s^–1^.

After the hot forging process, the specimens were cooled at four different cooling rates of 0.8 °C·s^–1^, 1.0 °C·s^–1^, 1.8 °C·s^–1^, and 2.9 °C·s^–1^, using a controlled compressed air-cooling system. A nonforged sample was monitored by a type K thermocouple to confirm the cooling conditions for each analysis. Experimental curves of temperature as a function of time were obtained, as depicted in Figure 1. The cooling rates were selected to meet the cooling conditions applied in industry, corresponding to intermediate and severe cooling conditions. Another aspect was related to the limits for obtaining a ferritic-pearlitic microstructure after cooling, as predicted by the CCT diagram of 38MnVS6 steel shown in Figure 1. For each condition, 7 hot-forged specimens and 1 nonforged specimen were analyzed. The 7 hot-forged specimens were tested for verifying the reproducibility of the results, while the nonforged specimen was used as a reference for the results.

**Figure 2:**
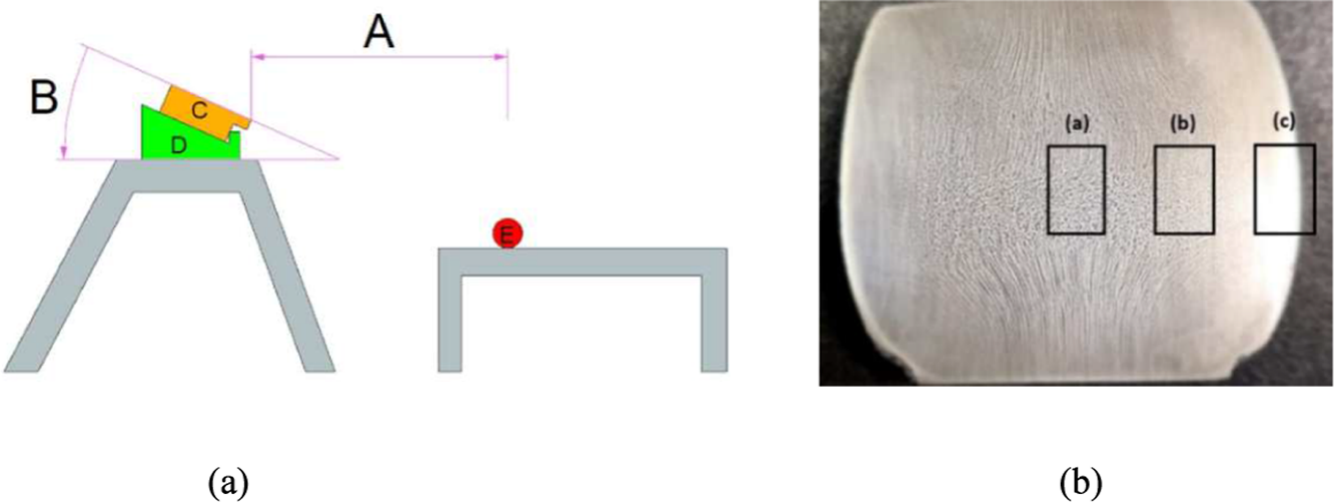
(a) Schematic representation of the cooling system; (b) longitudinal macrostructure and locations for microstructure analysis.

A system using compressed air with cooling rates varying according to the distance from the device to the specimens was developed. Figure 2a shows a schematic representation of the cooling system, where “A” is the distance between the compressed air outlet and the sample, “B” is the angle of inclination of the compressed air gun, “C” exemplifies the positioning of the compressed air gun, “D” is the device for tilting the compressed air gun, and “E” is the sample positioned for cooling. The distance “A” and the angle “B” are correlated to the defined cooling rates. At the end of the cooling step, a nonforged specimen and a hot-forged specimen for each cooling condition were sectioned, and the macrostructure at the central region was analyzed, as shown in Figure 2b. For microstructure analysis, the samples were ground by using SiC abrasive sandpapers (#180, #240, #320, #400, #600, and #120 grit), polished with alumina powder suspensions (1.0 and 0.3 μm), and finally etched with iodine reagent. Analysis was conducted using an optically inverted microscope (Olympus, PMG 3). The locations where microstructures were analyzed, identified as (a) core, (b) midradius, and (c) near-surface are also shown in Figure 2b.

The remaining samples were machined according to the ASTM E8 standard method for tensile tests^17^ for type 2 specimen, with a diameter of 9.0 ± 0.1 mm, a gauge length of 45.0 ± 0.1 mm, a reduced section length of 54 mm, and a fillet radius of 8 mm. A statistical approach based on the analysis of variance (ANOVA) using Minitab software was applied to analyze the main results of the experiments.

## 3 Results and Discussion

**Figure 3:**
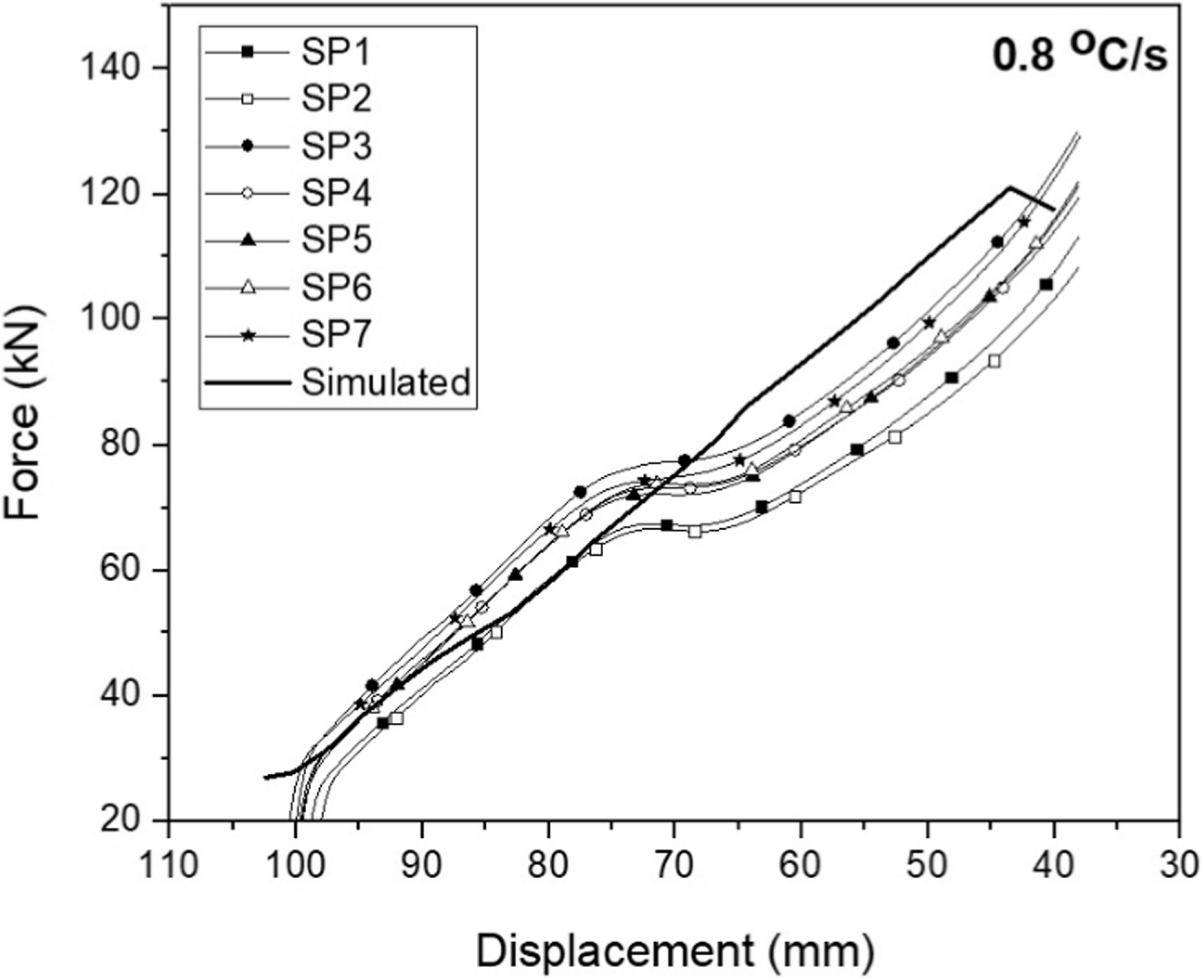
Simulated and experimental load curves as a function of displacement during forging (SP—specimen).

**Figure 4:**
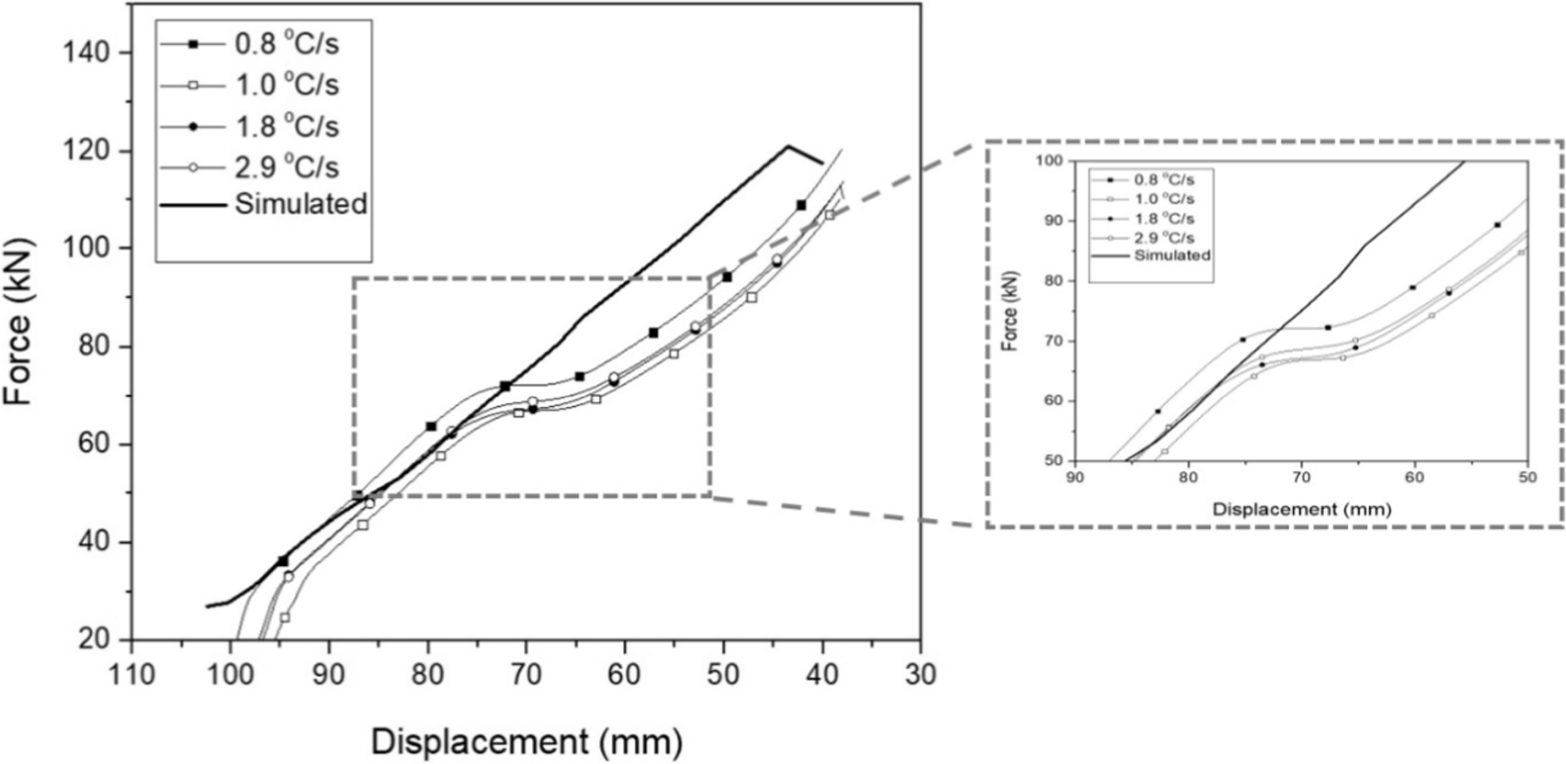
Average values of forging load as a function of displacement during deformation of specimens, which were subsequently cooled at cooling rates of 0.8, 1.0, 1.8, and 2.8 °C·s^–1^.

The numerical simulation results to predict the behavior of 38MnVS6 steel during hot semiclosed-die forging were validated against experimental data using the load–displacement curves. Figure 3 shows the curves of the first set of 7 hot-forged and cooled specimens at a cooling rate of 0.8 °C·s^–1^. For the other cooling conditions, Figure 4 presents the average values of the forging load as a function of the displacement during deformation of specimens as well as the comparison with the simulated curve. With these curves, it was possible to validate the results obtained via numerical simulation for the final forging load values. The simulated maximum load is in good agreement with the experimental values, with differences of less than 5%.

The simulation satisfactorily represents the behavior of the material during plastic deformation. However, after 75 mm of displacement, the material presented dynamic recrystallization, with the formation of strain-free grains, softening the material, distorting the curve, and deviating from that predicted by the numerical analysis. This region is shown in detail in Figure 4. Thus, more accurate simulations can be achieved if the dynamic recrystallization behavior can be modeled using approaches such as comparison between theoretical-experimental data and data obtained from databases.

**Figure 5:**
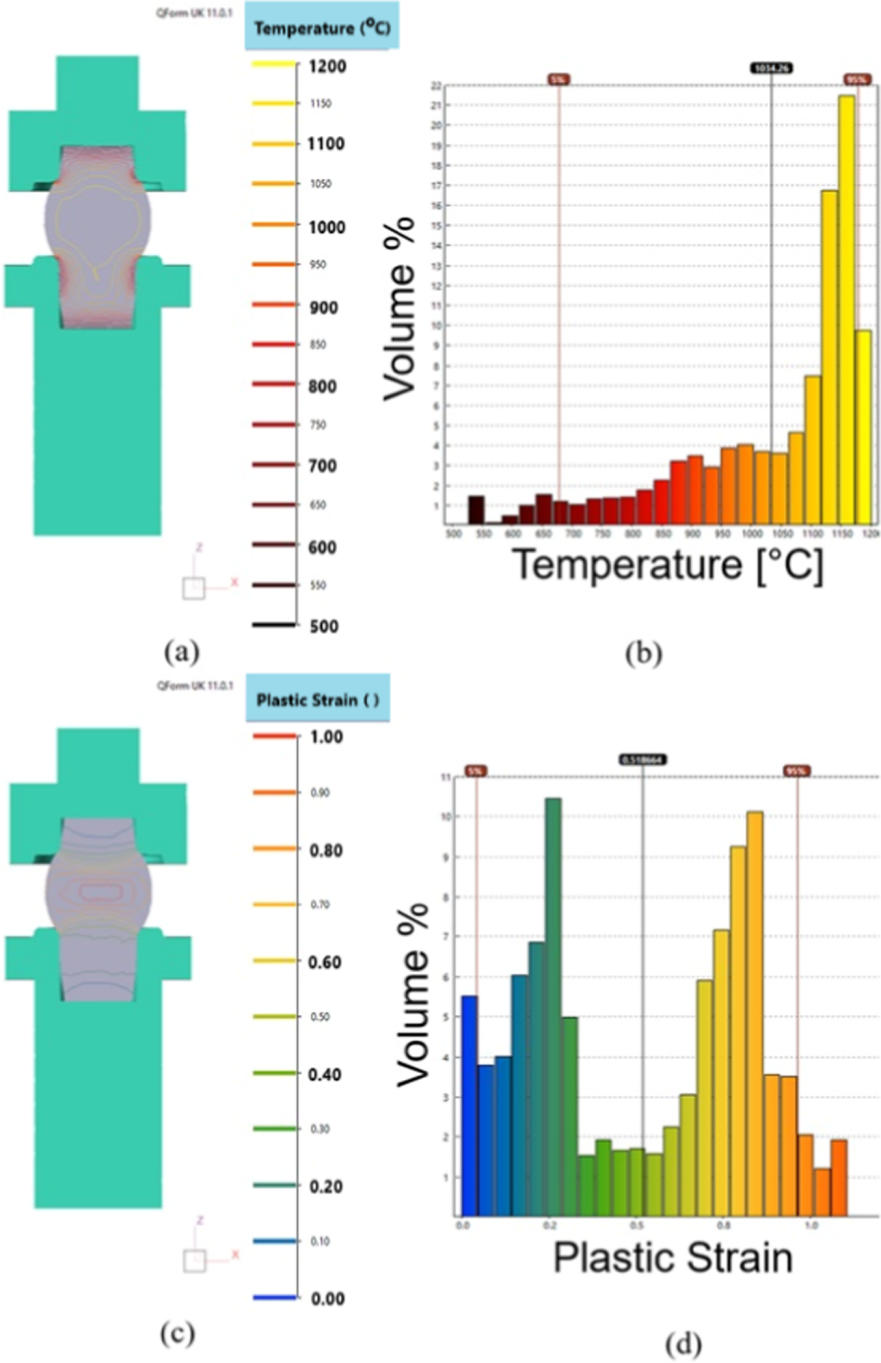
(a) Temperature distribution in volume, (b) temperature distribution histogram, (c) plastic deformation distribution in volume, and (d) deformation distribution histogram.

Regarding the temperature distribution in volume, Figure 5a,b shows that the largest portion of the sample has temperatures above 1035 °C, with lower temperatures on the surfaces in the regions in contact with the tools. Observing Figure 5c,d, it is possible to see that the greatest deformations occurred in the region external to the tools, with values above 90%, while the internal regions of the tools deformed by less than 10%. This behavior demonstrates that the experimental apparatus can be used to simulate a hot forging process in a semiclosed die.

**Figure 6:**
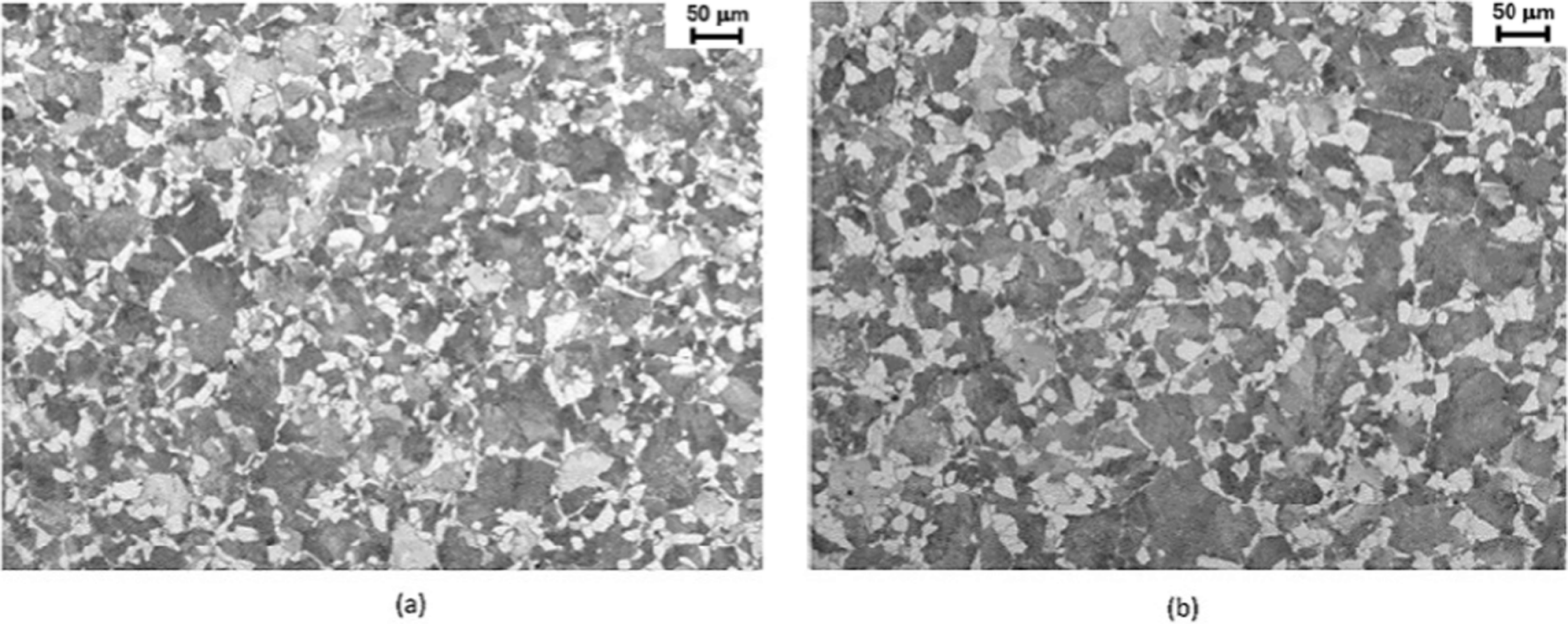
Microstructure of the as-received 38MnVS6 steel: (a) midradius and (b) core regions.

Micrographs of the as-received material (hot-rolled) are shown in Figure 6. The observed microstructure reveals the presence of ferrite and pearlite. At the midradius region, a thin continuous ferrite network showing a grain thickness of 3–4 μm was observed. The other ferrite morphology comprises coarser grains, adjacent to pearlite grains, of 10–12 μm size. The size of the pearlite grains is quite coarse of around 40–45 μm. The volume fractions of pearlite and ferrite were about 70 and 30%, respectively, as estimated by ImageJ software.

**Figure 7:**
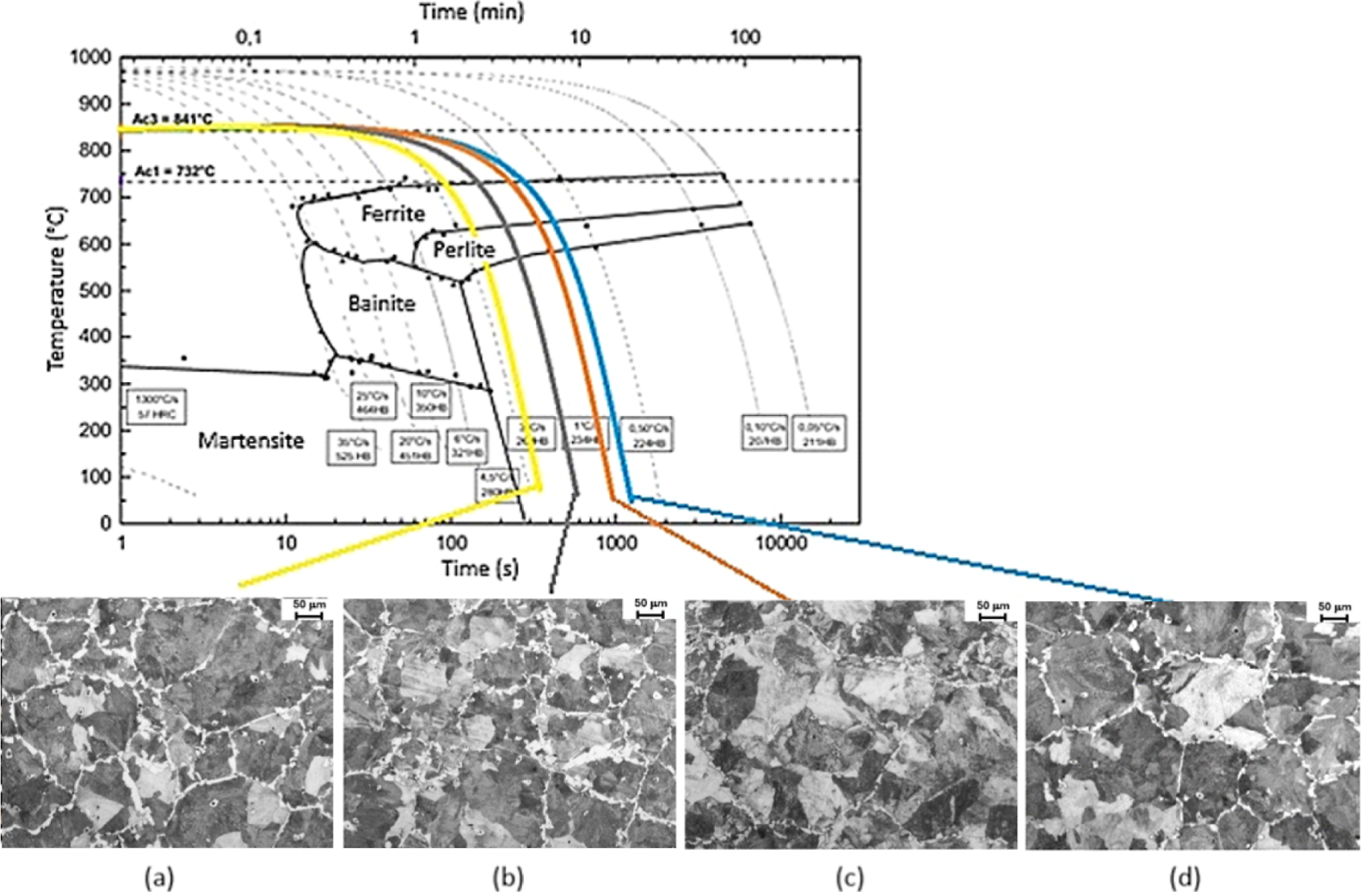
CCT diagram of 38MnVS6 steel with microstructures in the midradius zone of nonforged specimens cooled at (a) 2.9 °C·s^–1^, (b) 1.8 °C·s^–1^, (c) 1.0 °C·s^–1^, and (d) 0.8 °C·s^–1^.

Analysis of the microstructures of the nonforged samples were carried out, categorizing the material according to the subjected cooling rates, as shown in Figure 7.

**Figure 8:**
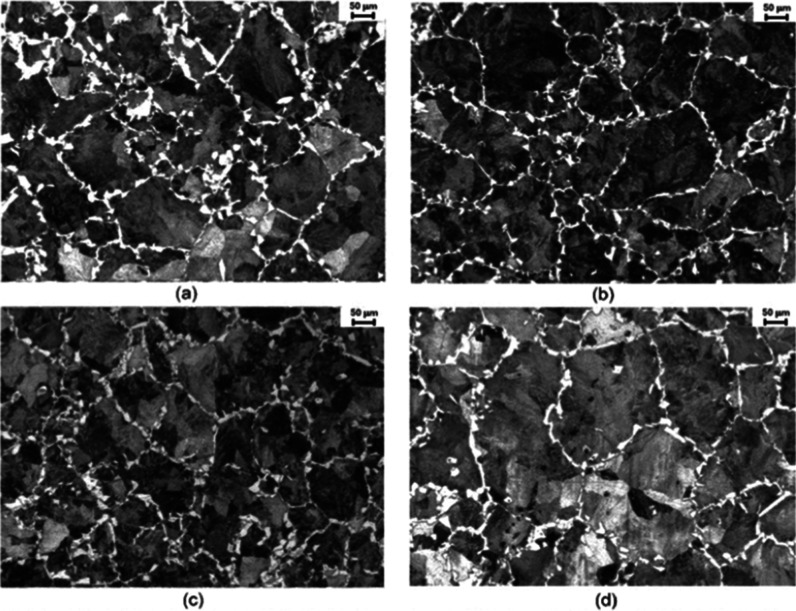
Core microstructures of hot-forged specimens for each cooling rate: (a) 0.8 °C·s^–1^, (b) 1.0 °C·s^–1^, (c) 1.8 °C·s^–1^, and (d) 2.9 °C·s^–1^.

**Figure 9:**
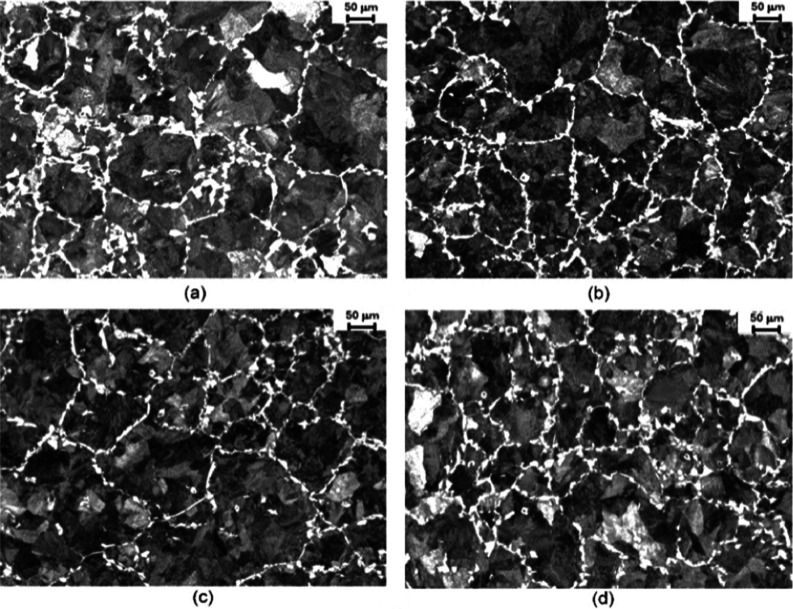
Midradius microstructures of hot-forged specimens for each cooling rate: (a) 0.8 °C·s^–1^, (b) 1.0 °C·s^–1^, (c) 1.8 °C·s^–1^, and (d) 2.9 °C·s^–1^.

Figures 8 and 9 present the microstructures obtained at the core and midradius regions, as illustrated in Figure 2b, for the hot-forged specimens. Regarding microstructure, Erçayahn et al.^15^ establish that for temperature of heating above 900 °C was found ferrite-pearlite, with ferrite concentrated on grain boundaries and pearlite focused on core of grains. Hiremath et al.^20^ observed that for 38MnVS6 steel forged at 800 °C, 850 °C, 900 °C, and 1050 °C and cooled at the rates of 0.75 °C·s^–1^ and 1.5 °C s^–1^, pearlite content decreased, and ferrite content increased as the forging temperature increased until 900 °C due to microstructure refinement. It is important to highlight that despite the different cooling rates, all samples presented a microstructure composed of pearlite surrounded by ferrite at the grain boundaries. This behavior was also observed by Hiremath et al.^20^ and Erçayahn et al.^15^ According to Holappa et al.,^21^ the predominant microstructure of 38MnSi6 steel is pearlite, which agrees with this study. It is important to highlight that there were no significant differences in the resulting microstructure regarding the presence of the pro-eutectoid phase and the pearlite microconstituent for the different cooling conditions analyzed. According to Bangaru and Sachdev,^22^ during ferrite transformation under air cooling conditions, the formation of a large amount of carbonitrides in bands in the microstructure is expected.

In the microstructures, the average linear lengths of the grain sizes were measured and correlated to the ASTM number, with the results presented in Table 5. The average linear grain length of the as-received material (hot-rolled) was about 68 ± 3 μm at the core and midradius. The maximum and minimum values were 228 and 154 μm for cooling rates of 0.9 °C·s^–1^ and 2.9 °C s^–1^, respectively, for the cooled samples (identified as [n] in Table 5) at the core, whereas for the hot-forged condition (identified as [d] in Table 5), the average linear grain length decreased at the core, ranging from 117 μm for samples cooled at 2.9 °C s^–1^ to 131 μm for samples cooled at 0.8 °C s^–1^.

**Table 5:** Medium Grain Size at the Core and Midradius Regions of the Specimens

	core		midradius	
cooling rate (°C·s^–1^)	medium linear length (μm)	ASTM grain size	medium linear length (μm)	ASTM grain size
as received	66	5	71	5
0.8 [n]^a^	228	1	224	1
1.0 [n]	229	1	223	1
1.8 [n]	173	1	238	1
2.9 [n]	154	1	228	1
0.8 [d]	131	3	141	2
1.0 [d]	125	3	146	2
1.8 [d]	119	3	162	1
2.9 [d]	117	3	146	2

a [n] nonforged condition; [d] hot-forged condition.

**Figure 10:**
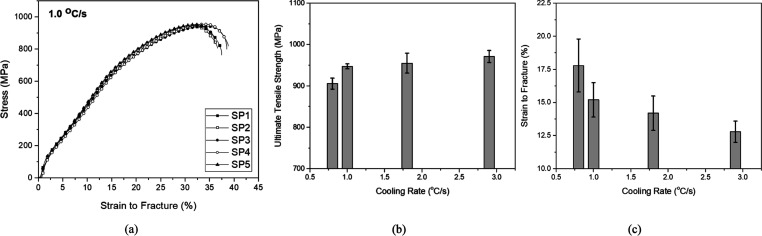
(a) Stress vs strain curves for 1.0 °C·s^–1^, (b) UTS, and (c) strain to fracture.

For tensile strength analyses, specimens from each cooling rate were tested, and an example of the results for 1.0 °C·s^–1^ is shown in Figure 10a. For each analysis, results from 5 specimens were considered. With the results, it was possible to obtain information regarding the average strain to fracture and ultimate tensile strength (UTS) of the steel as well as standard deviation values, as shown in Figure 10b,c.

It is possible to control the properties of steel by controlling the grain size. Improvement of mechanical strength can be achieved by reducing grain size, where grain boundaries act as barriers to the movement of dislocations, as investigated by Pluphrach^23^ on the effect of vanadium and niobium additions on the microstructure and mechanical properties of a microalloyed HSLA steel. In addition to grain refining, Skobir^24^ discussed two other hardening mechanisms for microalloyed steels: solid solution and the formation of stable nitrides, with the relative contribution of each determined by the chemical composition and thermal, thermomechanical treatment processes, and others.

The results are in agreement with those founded by Hiremath et al. (2020)^20^ for 38MnVS6 steel regarding UTS, under controlled cooling after forging condition. For specimens of 38MnVS6 obtained on crankshaft part, Kousadikar and Gaunkar (2014)^18^ found 880 MPa for UTS. For the condition after forging but without cooling control, Siwiec et al. (2020)^19^ showed results between 808 and 867 MPa for UTS. According to Tsai et al.,^14^ the cooling conditions after hot forging control the ferrite-pearlite transformation and the precipitation of vanadium carbides, which directly influence the mechanical properties of microalloyed steels. The authors suggested that the grain size of prior austenite is fundamental for obtaining an optimized microstructure after forging. In the work reported by Show et al.,^25^ the results showed that the UTS obtained after controlling the forging cooling was better than that observed in the normalized condition for DMR-249A vanadium microalloyed steel. Similar behavior was related by Crowther and Li^26^ in their review about HSLA steels microalloyed with V. Similar results were reported by Crowther and Li^26^ in their review on the use of V in HSLA steels, mainly for steels forged at lower temperatures, resulting in increased strength due to grain refinement. Wang et al.^27^ also concluded that tensile strength increases substantially with increasing vanadium content in medium C steels, due to the precipitation of V(C, N) particles. These particles act by reducing the size of the austenite grains and pearlite colonies in addition to modifying the morphology of the ferrite.

**Figure 11:**
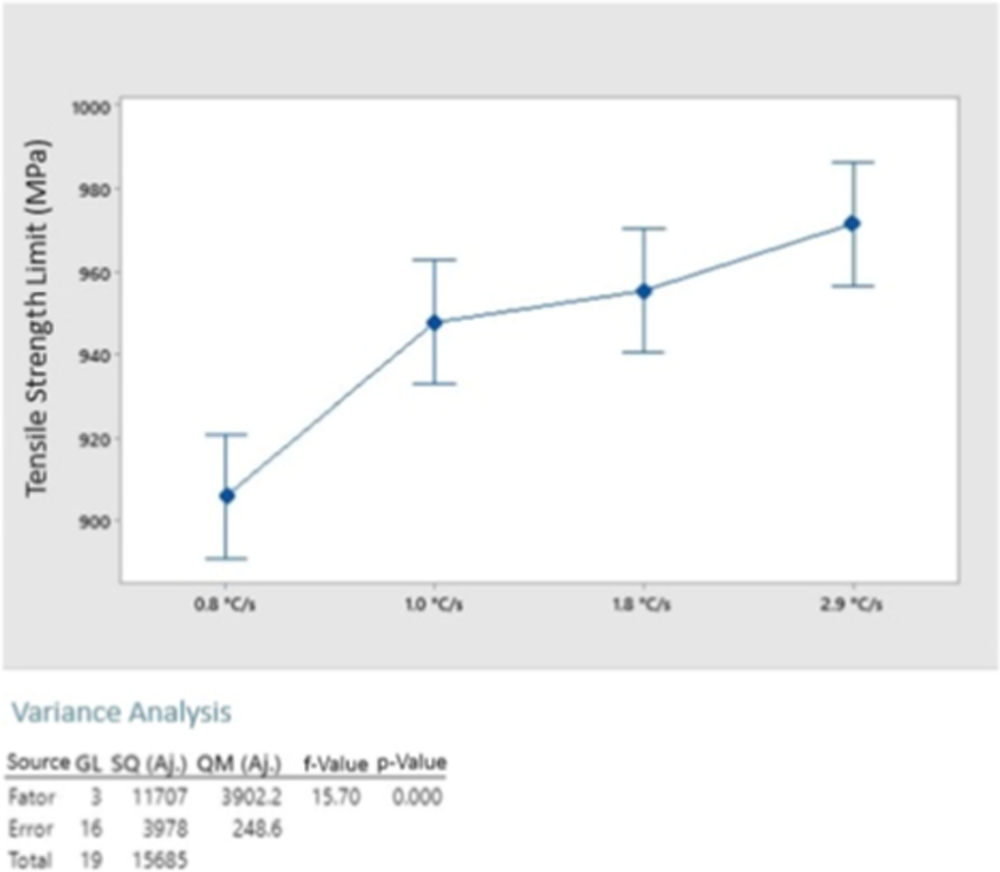
ANOVA results for UTS.

For a more in-depth analysis regarding the UTS, an ANOVA study was conducted. The first step involved a normal probability analysis, where the set of UTS results exhibited a normal behavior with 95% confidence, allowing for proceeding to the equality of the variance test, presented in Figure 11. Resulting in a *p*-value of 0.000 for the UTS study, it is possible to conclude with 95% confidence that the means found are different, and the UTS increases as the cooling rate is increased. These results agree with those reported by Equbal et al.^6^

## 4 Conclusions

This work investigatedthe influence of hot deformation and different cooling rates on themicrostructural formation of DIN 38MnVS6 microalloyed steel. The followingconclusions can be drawn from the results:

- using experimental and finite elementsimulation data, characteristics of both the material and the processwere analyzed, which were used to define the forging loads and inthe design of the dies,
- the results showed that the average linear grain length of the as-receivedmaterial was about 68 ± 3 mm at the core and midradius, whilethe cooled material showed values of 154 μm (at the core) and228 μm (at the midradius) for the highest cooling rate (2.9°C s^–1^). It was observed that the average lineargrain length in midradius remained similar despite varying coolingconditions,
- when hotforging was applied, the average linear length was 131 μm forsamples cooled at the lowest cooling rate (0.8 °C•s^–1^) and 117 μm at the highest cooling rate (2.9°C•s^–1^), a difference of 12%;
- higher cooling rates inducedhigher tensile strength due to grain refinement. Statistically, itis possible to conclude that there are differences between the resultsobtained for the cooling rate of 0.8 °C·s^–1^ when compared to the rates of 1.0 °C·s^–1^, 1.8 °C·s^–1^, and 2.9 °C·s^–1^.
